# Prevalence of Malocclusions and Associated Factors inBrazilian Children and Adolescents with CerebralPalsy: A Multi-Institutional Study

**DOI:** 10.1155/2020/8856754

**Published:** 2020-08-29

**Authors:** Andreia Medeiros Rodrigues Cardoso, Clara Regina Duarte Silva, Lays Nóbrega Gomes, Mariana Marinho Davino de Medeiros, Wilton Wilney Nascimento Padilha, Alidianne Fábia Cabral Cavalcanti, Alessandro Leite Cavalcanti

**Affiliations:** ^1^Department of Dentistry, Faculty of Dentistry, State University of Paraiba, Avenida Das Baraunas, S/N, Bodocongo, Campina Grande 58429-500, PB, Brazil; ^2^Department of Dentistry, Faculty of Dentistry, Federal University of Paraiba, Cidade Universitária, S/N, Castelo Branco, João Pessoa 58051-900, PB, Brazil

## Abstract

**Background:**

To assess the prevalence and factors associated with malocclusions in children and adolescents with cerebral palsy (CP).

**Methods:**

The study included 134 subjects with CP aged 2–18 years enrolled in six rehabilitation institutions and their caregivers, which provided demographic, systemic, and behavioral data. A calibrated researcher held oral examinations with record of the following malocclusion indexes, DAI and DMFT. Poisson regression analysis was used (*α* < 0.05).

**Results:**

About 85.8% (*n* = 115) of patients had malocclusion. In deciduous and mixed dentition (*n* = 99), increased overjet (75.8%), open bite (51.5%), posterior cross bite (19.2%), and anterior cross bite (3.0%) were identified. Increased overjet was associated with the age group of 2–5 years and mild communication impairment. Anterior open bite was more common in children who underwent tongue interposition, lip interposition, and pacifier sucking. Communication skills, mouth breathing, and tongue interposition were associated with posterior cross bite. Severe malocclusions (DAI > 30) were observed in 88.6% of patients with permanent dentition (*n* = 35) and were associated with liquid diet consistency and finger sucking.

**Conclusion:**

The prevalence of malocclusion in individuals with CP was high and associated with demographic, behavioral, and systemic factors.

## 1. Introduction

Cerebral palsy (CP) is a clinical condition with global prevalence of 2.1 per 1,000 live births [1, 2], defined as a group of developmental disorders of movement and posture of nonprogressive order, whose etiology involves brain injury that occurs during fetal development or in the infant's brain and may involve various areas of the brain, thus determining different clinical conditions [3–5].

Motor dysfunction in the head and neck region of individuals with CP can cause hypoxemia, contractures in the temporomandibular joint, vomiting, and aspiration pneumonia associated with gastroesophageal reflux, feeding difficulties, changes in facial growth, changes in the head and neck posture, drooling, and communication difficulties [4, 5]. In addition to motor disorders, patients with CP often have sensation, cognition, perception, behavior, and convulsive alterations [4, 5].

Malocclusion is one of the most frequent oral diseases in children and adolescents with CP, and the degree of mental impairment is directly proportional to the malocclusion severity [6–8]. Some studies have reported increased prevalence of malocclusion in subjects with CP when compared to population without this systemic condition [9, 10]. Individuals with CP are more likely to have parafunctional habits and mouth breathing, factors that may be directly associated with the development of malocclusion in these patients [11, 12].

There are some studies with individuals with CP in the literature assessing aspects related to dental caries [10, 13–16], periodontal condition [10, 16], and dental trauma [10, 17, 18]. Regarding malocclusion, there are only few studies analyzing its prevalence [9–11, 15, 19] and the association of this disease with demographic and socioeconomic factors [9, 12], nonnutritive sucking habits [11], type of breathing, and orofacial motor disorders [7, 9] in pediatric populations with special needs or not, and no studies assessing adjustment for socioeconomic, behavioral, systemic, and oral information exclusively in CP patients have been found in the literature.

Nevertheless, the presence of malocclusions has caused a negative impact on the quality of life of affected individuals and their families [20]. Thus, in order to serve as a diagnostic for the planning of health promotion actions aimed at CP patients, the aim of this study was to evaluate the prevalence of malocclusion in Brazilian children and adolescents with CP and its associated factors.

## 2. Materials and Methods

### 2.1. Study Population

This cross-sectional study was carried out in the city of João Pessoa, state of Paraiba, Brazil. The city has approximately 791,438 inhabitants and Human Development Index of 0.76 [21]. Data collection was held at the institution that provides therapeutic and educational activities and social integration for people with CP, in the city of João Pessoa, specifically, the Integrated Foundation for the Support of Disabled People, Association of Parents and Friends of Mentally Retarded Children, Helena Holanda Special Activities Center, Pestalozzi Association of Paraíba, Hippotherapy Association of Paraíba and Institute of Blind People of Paraiba.

The study population was composed of 166 children and adolescents aged 2–18 years and their caregivers. However, 134 children and adolescents (77 males and 57 females) actually participated in the survey, constituting a response rate of 80.72%. Of the 32 losses, eight refused to participate and 24 did not attend clinical examinations after three attempts.

Inclusion criteria were as follows: children and adolescents aged 2–18 years with CP diagnosis (ICD-10 G80) and enrolled in the institution that provides therapeutic activities for people with CP in João Pessoa and primary caregivers older than 18 years of age, defined as responsible for making decisions and carrying out the daily activities of people with CP [14].

### 2.2. Ethical Aspects

Researchers followed the ethical guidelines recommended by the Brazilian and international law, and the study was approved by the Ethics Research Committee (CEP) of the State University of Paraíba (CAAE 20215413.4.0000.5187). All participants/caregivers signed the free and informed consent form.

### 2.3. Calibration and Training Process

Calibration consisted of two stages (theoretical and clinical) and was carried out by a standard gold dentist specialist in pediatric dentistry. In the theoretical stage, each criterion and code was discussed for the diagnosis of malocclusion [22–24] and dental caries [25]. The clinical phase for malocclusion and dental caries involved the clinical examination of 60 children without neurological impairment enrolled in preschool and public school, respectively. Kappa values to evaluate concordance for the diagnosis of dental caries and malocclusion were 0.75 and 1.00, respectively.

### 2.4. Data Collection

Data collection was performed by a single examiner and in a clinic waiting room of the institution. Initially, clinical data with information regarding CP location and type of neuromuscular dysfunction were obtained in each medical record of the sample in the institution. Then, a clinical form was filled after the performance of face-to-face inquiries with caregivers and oral clinical examination in children and adolescents with CP.

The instrument contained socioeconomic (sex and age of the child, caregiver schooling, and family income), behavioral (nonnutritive sucking habits, bottle feeding, and diet consistency), systemic (CP location, type of neuromuscular dysfunction, and type of breathing), and oral information (malocclusion, dental caries, lip hypotonia, tongue interposition, and lip interposition) of children and adolescents with CP. Caregivers answered the socioeconomic and behavioral questions.

Oral examinations were performed by the examiner with the patient seated in his own wheelchair or traditional chair after tooth brushing held under supervision [13]. The examiner used a LED-type lamp (light emitting diode) of 250 lumens coupled to the head, flat dental mirrors, millimeter probe (Community Periodontal Index (CPI), Trinity Ind. Com Ltd, São Paulo, SP, Brazil), mouth openers, wooden spatulas, disposable gauze, and individual protective equipment [25].

In general clinical examination, type of breathing [9, 18], presence of lip hypotonia [26], tongue interposition [11], and lip interposition were observed. In the intraoral clinical examination, DMFT index [25], malocclusion index [22, 23], and Dental Aesthetic Index (DAI) [24] were collected.

### 2.5. Statistical Analysis

Descriptive statistics were used to characterize the sample. The chi-squared and Fisher's exact tests were used to compare the distribution of subjects with moderate and severe malocclusion in permanent dentition according to the DAI criteria (*α* < 0.05). The bivariate and multivariate Poisson regression with robust variance was also used to determine the association between dependent variables (presence of overjet increased, anterior open bite, and posterior cross bite in deciduous and mixed dentition, as well as severe malocclusion-DAI >30 in the permanent dentition) and independent variables (socioeconomic, behavioral, systemic, and oral), after categorization (*α* < 0.05). Hierarchical procedure was used to select variables that have achieved the *p* value <0.20 in the bivariate analysis. The analysis was conducted at three levels, from distal to proximal determinants: (1) socioeconomic, (2) systemic, and (3) behavioral. Variables with *p* < 0.05 in the adjusted analysis were included in the final regression model. All tests were performed using the Statistical Package for Social Sciences software (SPSS for Windows, version 18.0, SPSS Inc, Chicago, IL, USA).

## 3. Results

The prevalence of malocclusion was 85.8% (*n* = 115) of children and adolescents with CP. Presence of canine angulation in class I (52.5%), increased overjet (75.8%), open bite (51.5%), and posterior cross bite (19.2%) in patients with deciduous and mixed dentition was observed (Table 1).

In the multivariate analysis with the distribution of patients with deciduous and mixed dentition for the presence of overjet increased, independent variables aged 2–5 years (PR = 0.72; 95% CI: 0.55–0.93) and mild communication problem (PR = 0.73; 95% CI: 0.55–0.96) were associated with the presence of overjet increased (*p* < 0.05; Table 2).

Anterior open bite in deciduous and mixed dentition was associated with the presence of tongue interposition (PR = 1.86; CI 95%: 1.01–3.43), lip interposition (RP = 1.46; CI 95%: 1.01–2.13), and no pacifier sucking (PR = 0.64; CI 95%: 0.47–0.88; Table 3). However, communication capability and mouth breathing were associated with presence of posterior cross bite, in the final model (*p* < 0.05; Table 3).

In the distribution of patients with permanent dentition (*n* = 35) by the DAI criterion, 88.6% of patients had very severe malocclusion (Table 1). The frequency of diastema (≥1 mm) and maxillary anterior overjet (≥4 mm) was higher in subjects with severe malocclusions, with significant differences (*p* < 0.05; Table 4). Severe malocclusion given by the DAI index was associated with solid consistency of diet (PR = 0.60; 95% IC: 0.36–0.99) and absent of digital sucking habit up to the first 24 months or more (PR = 0.57; 95% IC: 0.33–0.98) (Table 5).

## 4. Discussion

This study evaluated the prevalence of malocclusion in children and adolescents aged 2–18 years with CP and associated factors. This is one of the few studies conducted in Brazil and in the world investigating, only in these individuals, the association of the disease with socioeconomic, behavioral, systemic, and oral factors. Despite having used a nonrepresentative sample, it is noteworthy that the number of subjects with CP investigated (one-hundred and thirty-four) is much higher than samples from other Brazilian [9, 12, 15, 19] and foreign studies [7, 10].

The prevalence of malocclusion in the study population was 85.8%; lower or equivalent proportions were found by other studies carried out in Brazil [9, 15, 19], India [10], and Spain [7]. In the deciduous and mixed dentition, the most frequent injuries were increased overjet, open bite, posterior cross bite, and anterior cross bite, corroborating findings from studies of India [10] and Brazil [12]. The frequency of severe malocclusion in the permanent dentition was 88.6%; similar results were also found in Spain [7] and Brazil [9, 19]. These high malocclusion frequencies can be explained by the fact that children with CP present high prevalence of motor dysfunction in orofacial muscles [7–11, 15, 19], nonnutritive sucking habits [11, 12], and mouth breathing [7–10, 19].

In patients with CP who had deciduous and mixed dentition, increased overjet was the most observed alteration (75.8%), and this high prevalence has also been observed in other studies that evaluated children with CP [9, 10]. The frequency of increased overjet in children with CP was higher when compared to that of the control groups [9, 10]. The high prevalence in this group of patients is due to the changes of orofacial muscle tone, which consequently changes the patterns of facial growth and development [9, 10]. In addition, the inclination of the upper incisors is the result of the action of lips against the teeth, according to the theory of neuromuscular tissue stretch and the negative feedback mechanism, so in patients with CP, lip hypotonia does not allow mouth sealing and control of the jaw protrusion [7].

In this study, lip hypotonia was associated with the presence of increased overjet exclusively, in the bivariate analysis, according to a previous study [7]. However, in the final model, association of increased overjet with the age group of 2–5 years (PR = 0.72; 95% CI 0.55–0.93) and mild communication problem (PR = 0.73; 95% CI 0.55–0.96) was observed. Studies assessing the relationship between age and communication skills in these individuals have not been found in the literature. However, the older the child, the greater the exposure time to systemic and behavioral factors that affect the occlusion development.

The problems in the communication skills of patients with CP are due to the brain injury caused by the disease [4, 5]. Therefore, the greater the patient's motor and mental impairment, the greater the communication difficulty [4, 5]. Nevertheless, studies have shown a directly proportional relationship between motor impairment and malocclusion severity in patients with CP [6, 7], which justifies the higher probability of increased overjet in patients with communication difficulty.

In this study, the prevalence of anterior open bite in deciduous and mixed dentition was 51.5%, and its presence was associated with tongue interposition, lip interposition, and pacifier sucking up to the first 24 months of age or older. In a previous study, Brazilian children diagnosed with CP were three times more likely to have open bite than children with Down syndrome (DS) [12]. In addition, another study found a significant association between spastic type of CP and anterior open bite [27]. Thus, it is believed that this type of neuromuscular dysfunction reaches posture and movements of head and neck muscles, leading to increased spasticity of neck muscles, which causes the head to tilt backwards and lowers the jaw [11, 27]. Therefore, these muscle impairments can cause direct or indirect changes in the facial and occlusal development patterns, as they favor the presence of lip hypotonia, drooling, and systematic tongue anteriorization, facilitating the installation and maintenance of tongue interposition [11, 12, 17, 28].

In addition to the clinical condition of CP and spastic dysfunction, combination of nonnutritive sucking habits with open bite in Brazilian children with CP and DS was also found in another study [12]. The authors reported that the imbalance between external and internal muscle forces as well as the increase in mandible width and tendency to a reduction in jaw width may have contributed to this occurrence [11, 12].

Also in this study, the presence of posterior cross bite was evaluated in the final regression model with caregiver's educational level, type of disability, communication capability, type of breathing, lip hypotonia, diet consistency, tongue interposition, and digital sucking habit up to the first 24 months or more (*p* < 0.20). After analysis, the presence of posterior cross bite was significantly associated with children who performed mouth breathing and moderate communication problem (*p* < 0.05). Mouth breathing is very common in children with CP due to the presence of changes in the head and neck posture and/or obstruction and respiratory infections [7, 12], so that its presence has shown negative effects on growth and development of neurocranium and viscerocranium, which can result in malocclusion [29]. However, communication skills are directly related to the severity of motor impairment as reported for increased overjet.

A previous study also evaluated the factors associated with posterior cross bite in children with CP and DS [12] showed the association with children who used feeding bottle up to the first 24 months or more and who had nonnutritive sucking habits and respiratory infections [12]. The relationship of these three factors can be explained by the negative pressure that they cause inside the mouth and that interfere with the proper alignment of teeth and palate, which can lead to the development of posterior cross bite [11, 12, 29]. However, the literature also shows inconsistency on the association between bottle-feeding and malocclusion in children with and without CP [30], highlighting the need for more longitudinal studies to evaluate cause and effect in CP populations.

Malocclusion in permanent dentition was assessed by DAI [24]. DAI was adopted by the World Health Organization (WHO) in an attempt to establish a simple and universally acceptable orthodontic index for use in epidemiological surveys, which was considered valid and reliable for the determination of orthodontic treatment needs [24, 31]. In this evaluation, 88.6% of Brazilian children and adolescents with CP had severe malocclusion, similar to findings from other studies carried out in Spain [7] and Brazil [9, 19]. In bivariate analyses, severe malocclusion showed associated with age, caregiver schooling, diet consistency, lip interposition, feeding bottle, digital sucking, pacifier sucking, and loss of 1st permanent molar (*p* < 0.05; Table 5). However, exclusively, liquid diet consistency, and finger sucking after the first 24 months of age were associated with the presence of severe malocclusion in the permanent dentition in the final model.

Some studies on the association of severe malocclusion in children, adolescents, and adults with CP [7, 9], including children without CP [9], found the association with the presence of CP [9], mouth breathing [7, 9], lip hypotonia [7, 9], drooling [9], long facial type [9], and head hyperextension [7]. Given the above, motor and posture head and neck dysfunctions resulting from CP favors the need to feed through a liquid diet as well as the presence of several parafunctional habits that can alter facial and occlusal development patterns [7, 9, 11, 12, 27, 28, 32].

Therefore, the present study showed high prevalence of malocclusion and its association with demographic, systemic, and behavioral factors. However, due to the cross-sectional nature of this study, cause and effect relationships could not be performed. In addition, behavioral characteristics were obtained from reports of caregivers, which could be subject to memory biases [9, 12]. Nevertheless, the participants of this study were from five rehabilitation institutions and the results are therefore not necessarily representative of all individuals with CP. However, epidemiological studies with Brazilian individuals with CP with external validity were not found in the literature due to the absence of records in government databases. Therefore, longitudinal and case-control studies with probability samples should be carried out to better understand the etiology of malocclusion in populations with CP.

Nevertheless, pediatric dentists and orthodontists should recognize the need for their integration into multidisciplinary health team accompanying children and adolescents with CP even at an early age in order to intervene, together with professionals and caregivers, in the prevention and control of behavioral factors and those coming from motor and postural head and neck disorders to prevent and treat malocclusions in these individuals, while improving their development and quality of life.

## 5. Conclusion

The prevalence of malocclusion in Brazilian children and adolescents with CP was high and associated with demographic (age), behavioral (tongue interposition, lip interposition, pacifier sucking, and digital sucking habit), and systemic factors (communication capability, type of breathing, and diet consistency).

## Figures and Tables

**Table 1:** Distribution of children and adolescents with CP according to the presence of malocclusion and DAI.

Variables and attributes	*N*	%
*Canine angulation (n* *=* *99)*		
Class I	52	52.5
Class II	33	33.3
Class III	6	6.1
No information	8	8.1

*Overjet (n* *=* *99)*		
Normal	14	14.1
Increased	75	75.8
Top-to-top	2	2.0
Anterior cross bite	3	3.0
No information	5	5.1

*Overbite (n* *=* *99)*		
Normal	29	29.3
Low	4,0	4.0
Open bite	51	51.5
Deep	11	11.5
No information	4	4.0

*Posterior cross bite (n* *=* *99)*		
Absent	79	79.8
Present	19	19.2
No information	1	1.0

*DAI criteria (n* *=* *35)*		
No malocclusion (DAI < 25)	3	8.6
Defined malocclusion (DAI = 26–30)	0	0
Severe malocclusion (DAI = 31–35)	1	2.9
Very severe malocclusion (DAI > 36)	31	88.6

**Table 2:** Distribution of children of 2–12 years with CP in bivariate and multivariate Poisson regression models for the presence of overjet increased (>2 mm) and independent variables.

	Overjet increased		Bivariate		Multivariate	
	Absent, *n* (%)	Present, *n* (%)	*p* value	Not adjusted RP^*∗*^(CI 95%)	*p* value	AdjustedRP^*∗*^(CI 95%)
*Level 1*: *socioeconomic characteristics*						
*Gender*						
Male	18 (31.6)	39 (68.4)	0.040	0.79 (0.64–0.99)	—	—
Female	6 (14.3)	36 (85.7)		1.00	—	—

*Age (years)*						
2 to 5	15 (36.6)	26 (63.4)	0.029	0.75 (0.58–0.97)	0.015	0.72 (0.55–0.93)
6 to 12	9 (15.5)	49 (84.5)		1.00	—	1.00

*Caregiver's educational level*						
≤8 years	10 (38.5)	16 (61.5)	0.099	0.76 (0.55–1.05)	—	—
>8 years	14 (19.2)	59 (80.8)		1.00	—	—

*Family income (minimum wage* *=* *$266.70)*						
≤1 minimum wage	5 (17.2)	24 (82.8)	0.255	1.13 (0.91–1.41)	—	—
>1 minimum wage	19 (27.1)	51 (72.9)		1.00	—	—

*Level 2*: *systemic characteristics*						
*CP location*						
Tetraparesis	5 (16.1)	26 (83.9)	0.523	1.17 (0.71–1.92)	—	—
Diparetic	14 (28.0)	36 (72.0)	0.975	1.01 (0.61–1.66)	—	—
Hemiparesis	2 (28.6)	5 (71.4)		1.00	—	—

*Type of disability*						
Spastic	21 (24.1)	66 (75.6)	0.000	0.75 (0.67–0.85)	—	—
Athetoid	3 (30.0)	7 (70.0)	0.085	0.70 (0.46–1.05)	—	—
Mixed	0 (0.0)	1 (100.0)		1.00	—	—

*Communication capability*						
Normal	3 (18.8)	13 (81.2)	0.084	0.81 (0.64–1.02)	0.506	0.89 (0.65–1.23)
Mild deficiency	4 (26.7)	11 (73.3)	0.046	0.73 (0.54–0.99)	0.028	0.73 (0.55–0.96)
Moderate deficiency	17 (25.8)	49 (74.2)	0.000	0.74 (0.64–0.85)	0.324	0.82 (0.56–1.20)
Serious deficiency	0 (0.0)	2 (100.0)		1.00		1.00

*Type of breathing*						
Nasal	15 (38.5)	24 (61.5)	0.010	0.70 (0.53–0.91)	—	—
Mouth	7 (12.5)	49 (87.5)		1.00	—	—

*Diet consistency*						
Liquid	10 (23.8)	32 (76.2)	0.931	1.00	—	—
Solid	14 (24.6)	43 (75.4)		0.99 (0.79–1.24)	—	—

*Lip hypotonia*						
Absent	11 (45.8)	13 (54.2)		1.00	—	—
Present	13 (17.6)	61 (82.4)	0.032	1.52 (1.03–2.23)	—	—

*Level 3: behavioral characteristics*						
*Tongue interposition*						
Absent	7 (25.0)	21 (75.0)		1.00	—	—
Present	15 (22.4)	52 (77.6)	0.780	1.03 (0.80–1.32)	—	—

*Lip interposition*						
Absent	22 (28.2)	56 (71.8)		1.00	—	—
Present	1 (5.9)	16 (94.1)	0.040	1.31 (1.09–1.53)	—	—

*Digital sucking habit up to the first 24 months or more*						
Absent	23 (25.0)	69 (75.0)	1.00	1.00 (0.56–1.78)	—	—
Present	1 (25.0)	3 (75.0)		1.00	—	—

*Pacifier sucking habit up to the first 24 months or more*						
Absent	15 (24.2)	47 (75.8)	0.808	1.03 (0.80–1.31)	—	—
Present	9 (26.5)	25 (73.5)		1.00	—	—

^*∗*^Variables incorporated in the multivariate model of overjet increased (*p* < 0.20), gender, age, caregiver's educational level, type of disability, communication capability, type of breathing, lip hypotonia, and lip interposition. ^†^Poisson multivariate regression adjusted for the presence of overjet increased bite and socioeconomic, systemic, and behavioral characteristics (independent variables) by the hierarchical procedure.

**Table 3:** Distribution of children and adolescents with CP in Poisson bivariate and multivariate regression models for the presence of open bite and posterior cross bite and independent variables.

Variables	Open bite		Bivariate		Multivariate		Posterior cross bite		Bivariate		Multivariate	
	Absent, *n* (%)	Present, *n* (%)	Not adjusted RP^*∗*^		Adjusted RP^†^		Absent, *n* (%)	Present, *n* (%)	Not adjusted RP^*∗*^		Adjusted RP^†^	
			*p* value	95% CI	*p* value	95% CI			*p*-value	95% CI	*p*-value	95% CI
*Level 1: socioeconomic characteristics*												
*Gender*												
Male	27 (47.4)	30 (52.6)	0.797	1.05 (0.71–1.55)	—	—	45 (80.4)	11 (19.6)	0.941	1.00	—	—
Female	21 (50.0)	21 (50.0)	—	1.00	—	—	34 (81.0)	8 (19.0)	—	0.99 (0.81–1.20)	—	—

*Age (years)*												
2 to 5	19 (46.3)	22 (53.7)	0.718	1.07 (0.73–1.57)	—	—	35 (85.4)	9 (14.6)	—	1.00	—	—
6 to 12	29 (50.0)	29 (50.0)	—	1.00	—	—	44 (77.2)	13 (22.8)	0.298	1.10 (0.91–1.33)	—	—

*Caregiver's educational level*												
≤8 years	11 (42.3)	15 (57.7)	0.445	1.17 (0.78–1.75)	—	—	17 (65.4)	9 (34.6)	—	1.00	—	—
>8 years	37 (50.7)	36 (49.3)	—	1.00	—	—	62 (86.1)	10 (13.9)	0.067	0.75 (0.56–1.01)	—	—

*Family income (minimum wage* *=* *$266.70)*												
≤1 MW	11 (37.9)	18 (68.1)	0.153	1.31 (0.90–1.92)	—	—	25 (86.2)	4 (13.8)	—	1.00	—	—
>1 MW	37 (52.9)	33 (47.1)	—	1.00	—	—	54 (78.3)	15 (21.7)	0.322	1.10 (0.91–1.33)	—	—

*Level 2: systemic characteristics*												
*CP location*												
Tetraparesis	10 (32.3)	21 (67.7)	0.627	1.18 (0.59–2.35)	—	—	21 (70,0)	9 (30.0)	0.984	1.01 (0.72–1.38)	—	—
Diparetic	29 (58.0)	21 (42.0)	0.402	0.73 (0.35–1.50)	—	—	43 (86,0)	7 (14.0)	0.299	0.81 (0.55–1.19)	—	—
Hemiparesis	3 (42.9)	4 (57.1)	—	1.00	—	—	6 (85,7)	1 (14.3)	—	1.00	—	—

*Type of disability*												
Spastic	42 (48.3)	45 (51.7)	<0.001	0.51 (0.42–0.63)	—	—	69 (80.2)	17 (19.8)	0.158	0.80 (0.58–1.09)	—	—
Athetoid	6 (60.0)	4 (40.0)	0.018	0.40 (0,18–0.85)	—	—	8 (80.0)	2 (20.0)	—	1.00	—	—
Mixed	0 (0.0)	1 (100.0)	—	1.00	—	—	1 (100.0)	0 (0.0)	<0.001	0.81 (0.72–0.89)	—	—

*Communication capability*												
Normal	14 (87.5)	2 (12.5)	0.152	0.25 (0.03–1.66)	—	—	16 (100.0)	0 (0.0)	0.002	0.75 (0.64–0.87)	0.000	0.61 (0.47–0.78)
Mild deficiency	7 (46.7)	8 (53.3)	0.931	1.06 (0.24–4.61)	—	—	13 (86.7)	2 (13.3)	0.158	0.86 (0.71–1.05)	0.026	0.71 (0.52–0.96)
Moderate deficiency	26 (39.4)	40 (60.6)	0.788	1.21 (0.29–4.91)	—	—	48 (73.8)	17 (26.2)		1.00	—	1.00
Serious deficiency	1 (50.0)	1 (50.0)		1.00	—	—	2 (100.0)	0 (0.0)	0.000	0.73 (0.63–0.85)	0.013	0.62 (0.43–0.90)

*Type of breathing*												
Nasal	27 (69.2)	12 (30.8)	0.001	0.44 (0.26–0.73)	—	—	39 (94.7)	2 (5.3)	—	1.00	—	1.00
Mouth	17 (30.4)	39 (69.6)	—	1.00	—	—	40 (71.4)	16 (28.6)	0.002	1.32 (1.10–1.59)	0.017	1.25 (1.04–1.51)

*Lip hypotonia*												
Absent	19 (79.2)	5 (20.8)	—	1.00	—	—	23 (100.0)	0 (0.0)	<0.001	0.74 (0.65–0.85)	—	—
Present	28 (37.8)	46 (62.2)	0.007	2.98 (1.34–6.64)	—	—	55 (74.3)	19 (25.7)		1.00	—	—

*Diet consistency*												
Liquid	15 (35.7)	27 (64.3)	0.029	1.00	—	—	31 (73.8)	11 (26.2)	0.162	1.16 (0.94–1.43)	—	—
Solid	33 (57.9)	24 (42.1)	—	0.65 (0.44–0.95)	—	—	48 (85.7)	8 (14.3)	—	1.00	—	—

*Level 3: behavioral characteristics*												
*Tongue interposition*												
Absent	20 (71.4)	8 (28.6)	0.012	1.00	—	1.00	27 (96.4)	1 (3.6)	0.001	0.75 (0.64–0.88)	0.023	0.82 (0.70–0.97)
Present	25 (37.3)	42 (62.7)	—	2.19 (1.18–4.05)	0.047	1.86 (1.01–3.43)	48 (72.7)	18 (27.3)	—	1.00	—	1.00

*Lip interposition*												
Absent	40 (51.3)	38 (48.7)	—	1.00	—	1.00	62 (79.5)	16 (20.5)	0.779	1.03 (0.80–1.32)	—	—
Present	5 (29.4)	12 (70.6)	0.057	1.44 (0.98–2.12)	0.049	1.46 (1.01–2.13)	14 (82.4)	3 (17.6)	—	1.00	—	—

*Feeding bottle up to the first 24 months or more*												
Absent	10 (58.8)	7 (41.2)	0.424	0.78 (0.42–1.43)	—	—	13 (76.5)	4 (23.5)	—	1.00	—	—
Present	34 (47.2)	38 (52.8)	—	1.00	—	—	58 (81.7)	13 (18.3)	0.651	0.93 (0.70–1.24)	—	—

*Digital sucking habit up to the first 24 months or more*												
Absent	44 (47.8)	48 (52.2)	0.933	1.04 (0.38–2.83)	—	—	72 (79.1)	19 (20.9)	<0.001	1.00	—	—
Present	2 (50.0)	2 (50.0)	—	1.00	—	—	4 (100.0)	0 (0.0)	—	0.79 (0.71–0.87)	—	—

*Pacifier sucking habit up to the first 24 months or more*												
Absent	35 (56.5)	27 (43.5)	0.019	0.64 (0.44–0.92)	0.006	0.64 (0.47–0.88)	50 (82.0)	11 (18.0)	0.537	1.00	—	—
Present	11 (32.4)	23 (67.6)	—	1.00	—	1.00	26 (76.5)	8 (23.5)	—	1.07 (0.86–1.33)	—	—

^*∗*^Poisson regression not adjusted for independent variables and presence of open bite and posterior cross bite. ^*∗∗*^Variables included in the multivariate model of open bite (*p* < 0.20), family income, type of disability, communication capability, type of breathing, lip hypotonia, diet consistency, tongue interposition, lip interposition, and pacifier sucking habit up to the first 24 months or more. ^*∗∗*^Variables incorporated in the multivariate model of posterior cross bite (*p* < 0.20), caregiver's educational level, type of disability, communication capability, type of breathing, lip hypotonia, diet consistency, tongue interposition, and digital sucking habit up to the first 24 months or more. ^†^Poisson multivariate regression adjusted for the presence of open bite and posterior cross bite and socioeconomic, systemic, and behavioral characteristics (independent variables) by the hierarchical procedure.

**Table 4:** Distribution of children and adolescents with CP according to DAI criteria and severity malocclusion in permanent dentition.

DAI criteria	Severity malocclusion (*n* = 35)		
	Moderate (<30), *N* (%)	Severe (>30), *N* (%)	*p* value
*Loss of anterior tooth*			
0	3 (10.7)	25 (89.3)	0.365^#^
≥1	0 (0.0)	7 (100.0)	

*Crowding in the incisal segment*			
None	2 (9.1)	20 (90.9)	0.941^#^
One or two segments	1 (8.3)	11 (91.7)	

*Spacing in the incisal segment*			
None	1 (12.5)	7 (87.5)	0.651^#^
One or two segments	2 (7.4)	25 (92.6)	

*Diastema*			
0	3 (20.0)	12 (80.0)	0.036^#^
≥1 mm	0 (0.0)	20 (100.0)	

*Maxillary anterior misalignment*			
0	0 (0.0)	0 (0.0)	—
≥1 mm	3 (8.6)	32 (91.4)	

*Mandibular anterior misalignment*			
0	0 (0.0)	0 (0.0)	—
≥1 mm	3 (8.6)	32 (91.4)	

*Maxillary anterior overjet*			
<4 mm	2 (50.0)	2 (50.0)	0.029^#^
≥4 mm	1 (3.2)	30 (96.8)	

*Mandibular anterior overjet*			
0	3 (8.6)	32 (91.4)	—
≥1 mm	0 (0.0)	0 (0.0)	

*Anterior open bite*			
0	3 (17.6)	14 (82.4)	0.062^#^
≥1 mm	0 (0.0)	18 (100.0)	

^*∗*^Chi-squared test. ^#^Fisher's exact tests.

**Table 5:** Distribution of children and adolescents with CP models in Poisson bivariate and multivariate regression models for the presence of severe malocclusion (DAI > 30) and independent variables.

Variable	Malocclusion		Bivariate		Multivariate	
	Moderate, *n* (%)	Severe, *n* (%)	Not adjusted RP^*∗*^		Adjusted RP^†^	
			*p* value	(CI 95%)	*p* value	(CI 95%)
*Level 1: socioeconomic characteristics*						
*Gender*						
Male	3 (15.0)	17 (85.0)	0.422	0.91 (0.72–1.14)	—	—
Female	1 (6.7)	14 (93.3)		1.00	—	—

*Age*						
11 to 12 years	0 (0.0)	7 (100.0)	0.046	1.16 (1.00–1.35)	—	—
13 to 18 years	4 (14.3)	24 (85.7)		1.00	—	—

*Caregiver's educational level*						
≤8 years	4 (30.8)	9 (69.2)	0.047	0.69 (0.48–0.99)	—	—
>8 years	0 (0.0)	22 (100.0)		1.00	—	—

*Family income (minimum wage* *=* *$ 266.70)*						
≤1 minimum wage	3 (27.3)	8 (72.7)	0.145	0.75 (0.52–1.10)	—	—
>1 minimum wage	1 (4.2)	23 (95.8)		1.00	—	—

*Level 2*: *systemic characteristics*						
*CP location*						
Tetraparesis	0 (0.0)	12 (100.0)	0.085	1.50 (0.94–2.38)	—	—
Diparetic	1 (8.3)	11 (91.7)	0.205	1.37 (0.84–2.35)	—	—
Hemiparesis	3 (33.3)	6 (66.7)		1.00	—	—

*Communication capability*						
Normal	2 (28.6)	5 (71.4)	0.312	0.77 (0.48–1.26)	—	-
Mild deficiency	0 (0.0)	4 (100.0)	0.157	1.09 (0.96–1.23)	—	—
Moderate deficiency	2 (8.3)	22 (91.7)		1.00	—	—

*Type of breathing*						
Nasal	3 (27.3)	8 (72.7)	0.085	0.72 (0.50–1.04)	—	—
Mouth	0 (0.0)	22 (100.0)		1.00	—	—

*Diet consistency*						
Liquid	0 (0.0)	16 (100.0)	0.046	1.00	0.049	1.00
Solid	4 (21.1)	15 (78.9)		0.78 (0.62–0.99)	—	0.60 (0.36–0.99)

*Lip hypotonia*						
Absent	3 (42.9)	4 (57.1)	0.112	1.68 (0.88–3.21)	—	—
Present	1 (3.6)	27 (96.4)		1.00	—	—

*Level 3: behavioral and oral characteristics*						
*Tongue interposition*						
Absent	2 (16.7)	10 (83.3)	0.606	1.00	—	—
Present	2 (10.0)	18 (90.0)		1.08 (0.80–1.44)	—	—

*Lip interposition*						
Absent	4 (15.4)	22 (84.6)	0.046	1.00	—	—
Present	0 (0.0)	6 (100.0)		1.18 (1.01–1.39)	—	—

*Feeding bottle up to the first 24 months or more*						
Absent	0 (0.0)	9 (100.0)	0.084	1.18 (0.97–1.44)	—	—
Present	3 (15.8)	16 (84.2)		1.00	—	—

*Digital sucking habit up to the first 24 months or more*						
Absent	4 (13.3)	26 (86.7)	0.046	0.86 (0.75–0.99)	0.043	0.57 (0.33–0.98)
Present	0 (0.0)	4 (100.0)		1.00	—	1.00

*Pacifier sucking habit up to the first 24 months or more*						
Absent	4 (14.3)	24 (85.7)	0.046	0.85 (0.73–0.99)	—	—
Present	0 (0.0)	6 (100.0)		1.00	—	—

*Loss of 1st permanent molar*						
No	4 (14.8)	23 (85.2)	0.046	0.85 (0.72–0.99)	—	—
Yes	0 (0.0)	7 (100.0)		1.00	—	—

^*∗*^Poisson regression not adjusted for independent variables and presence of severe malocclusion ^*∗∗*^Variables incorporated in the multivariate model (*p* < 0.20), age, caregiver schooling, family income, CP location, communication capability, type of breathing, diet consistency, lip hypotonia, lip interposition, feeding bottle, digital sucking, pacifier sucking, and loss of 1st permanent molar. ^†^Poisson multivariate regression adjusted for the presence of severe malocclusion and socioeconomic, systemic, and behavioral characteristics (independent variables) by the hierarchical procedure.

## Data Availability

The data used to support the findings of this study will be made available from the corresponding author upon reasonable request (alessandrouepb@gmail.com).
